# Pharmacokinetic variables of medium molecular weight cross linked chitosan nanoparticles to enhance the bioavailability of 5-fluorouracil and reduce the acute oral toxicity

**DOI:** 10.1080/10717544.2021.1944398

**Published:** 2021-07-22

**Authors:** Aisha Sethi, Mahmood Ahmad, Tayyaba Huma, Waqas Ahmad

**Affiliations:** a Faculty of Pharmacy and Alternative medicines, the Islamia University of Bahawalpur, Bahawalpur, Pakistan; b Faculty of Pharmaceutical Sciences, Government College University, Faisalabad, Pakistan; c Akhuwat FIRST, Faisalabad, Pakistan

**Keywords:** Medium molecular weight chitosan, chemical cross-linking, controlled delivery, 5-fluorouracil, tolerability, acute oral toxicity

## Abstract

To prepare glutaraldehyde-based cross-linked medium molecular weight chitosan nanoparticles encapsulated with 5-Fluorouracil (5-FU), to overcome dosing frequency as well as reducing acute oral toxicity and poor bioavailability of the drug. Medium molecular weight chitosan nanoparticles (MMWCH-NPs) were prepared by reverse micelles method based on glutaraldehyde (GA) cross-linking and optimized by the process as well as formulation variables like a various drug to polymer ratio, cross-linker volumes, varying stirring speeds (rpm), different time of rotation/stirring, respectively and their effects on the mean particles size distribution and entrapment efficiency %EE and %LC of NPs. Characterization of formulations was done by FTIR studies, TEM, PXRD, TGA, Stability, and dissolution drug release studies were performed by dialysis bag technique at both pH (1.2 & 7.4) and acute oral toxicity studies in albino rabbits. The formulated nanoparticles showed a smooth morphology with smaller particle size distribution (230–550 nm), zeta potential (−15 to −18 mV) required to achieve enhanced permeation and retention effect (EPR), entrapment efficiency (%EE 12–59%). These NPs exhibited a controlled drug release profile with 84.36% of the drug over a period of 24 h. Drug release data were fitted to different kinetic models which predominantly followed Fickian diffusion mechanism (*R*^2^ = 0.972–0.976, *N* = 0.326–0.256). The optimized formulation (5-FU6) was observed under DSC/TGA, TEM. PXRD curves, FTIR, which confirmed thermal stability, structural integrity, amorphous state, compatibility between drug and polymer of optimized (5-FU6) as well as reduced acute oral toxicity in albino rabbits. Cross-linked medium molecular weight chitosan nanoparticles are nontoxic, well-tolerated therefore could be the future candidate for therapeutic effects as novel drug delivery carrier for anticancer drug(s).

## Introduction

Drug development and formulation are highly challenging, as more time and labor-consuming, expensive processes. Even most of the drugs could not pass through earlier stages of a clinical trial without producing therapeutic outcomes at the targeted site of action (DiMasi et al., 2003). So, a considerable amount of therapeutic agent is dispersed to the healthy tissues or organs so as to be not participated in the pathological phenomenon, resulting in harmful effects (Langer, 1998). Hence, developing a targeted drug delivery system (TDDSs) is used to, overcome these adverse events by releasing the drug or therapeutic agent at the specified targeted site of action. This might reduce toxicity by the improving therapeutic efficacy of bioactive agents by better biodistribution and pharmacokinetics, which ultimately enhance the therapeutic agent’s bioavailability (Gregoriadis, 1977; Poste & Kirsh, 1983).

Paul Ehrlich; was the first who proposed this idea almost a century ago for developing a drug or magic bullet that selectively demolish disease cells or tissues without harming the healthy cells or organs of the body (Vasir et al., 2005; Strebhardt & Ullrich, 2008). After that, various researchers over the last decades have extensively focused on the advancement of novel drug delivery systems (NDDSs), though some evolution has been made on this concept the transformation of Ehrlich’s idea into clinical practices has advanced by; nanomedicines and nanotechnology (Duncan, 2003). NDDS has comprised of three major components: a drug, a targeting moiety, and a carrier system. A therapeutic agent is either encapsulated via passive absorption or chemical conjugation into the carrier. As; the drug carrier system is significantly produced its effects on the pharmacokinetics and pharmacodynamics of the drug, so it must be selected with great care. A wide variety of materials, natural, semisynthetic or synthetic polymers, lipids, surfactants have been used as drug carriers (Duncan, 2006; Torchilin, 2008). Chitosan is a natural biodegradable, biocompatible, and nontoxic widespread polymer due to medical and pharmaceutical applications like a lubricant, suspending agent, thickening agent, chelating agent, binding agent, and stabilizing agent (Sampathkumar & Yarema, 2005; Liu et al., 2008). It also supports ion exchange and chromatography techniques (Kumar et al., 2004). Chitosan structural formula is a linear amino polysaccharide randomly distributed (1–4) linked D-glucosamine, N-acetyl-D-glucosamine units, found in the exoskeleton of lobster, crabs, and shrimp (Jeong et al., 2005).

The solubility of polymer (CH) is pH-dependent due to the amino group’s presence in its molecule. It is a dilemma for oral drug delivery (ODDS) in CH-NPs formed by electrostatic interaction between a polyion and counterions which become uneven in gastric fluid. This issue was resolved by irretrievable chemical cross-linking by cross-linker glutaraldehyde (GA) used at different concentrations to modulate the release of drugs from NPs. It demonstrated diffusion of the drug from cross-linked chitosan NPs in a controlled fashion (Lehr et al., 1992; Akbuga, 1995).

An antitumor drug having higher therapeutic efficacy is the 5-fluorouracil (5-FU). It was introduced in 1958 which was used against various solid tumors like colon, rectum, and breast cancer. Its conventional oral brand is showed erratic absorption through GIT. As 5-FU shows rapid gastrointestinal absorption, after oral administration, it yields peak blood levels between 15 and 60 min (Diasio & Lu, 1994). Additionally, 5-FU has distracted toxicity in normal healthy cells; fast metabolic reaction by dihydro-pyrimidine dehydrogenase (DPD) enzyme, prompt renal clearance, elevated digestive pain inhibit its application in the management of cancer. 80% orally administered 5-FU has been reported to metabolize in the liver and kidney (Gamelin et al., 1996; Schilsky et al., 1998) while detoxified and excreted as F-ß-alanine via urine. Hence, the half-life of 5-FU is shorter, that is, 8–20 min (Zhu et al., 2009). Intravenous administration of 5-FU for solid types of cancers demonstrates severe cytotoxic effects by many previous studies in the literature. As intravenous administration of 5-FU was disturbed microbial flora of GIT track so, accurately designed oral formulations of 5-FU using various biocompatible, biodegradable, smart pH sensitive polysaccharides have employed for sustained, controlled delivery of therapeutic agent(s), that is, depending upon the selection of polymer and networking pattern on tumor cells by most of the researchers in the literature as well. Cross-linking got attention due to drug loading (%LC) and entrapment efficiency (%EE) from NPs which affect therapeutic diffusion and tunable physicochemical properties of nano-carrier (NPs) to facilitate sustained but the controlled release of the chemotherapeutic agents at the specific targeted site without harming the other body tissues (Fournier et al., 2004; Zhang, Cheng, et al., 2008).

In this part of the research work, we intended to prepare, characterize and optimize formulations of medium molecular weight cross-linked chitosan NPs to find out the effect of different process and formulation variables for targeted deliverance of 5-FU through nano-carrier to reduced systemic toxicity as well as reduced dosing frequency which simultaneously release the drug in a controlled fashion and reduced acute oral toxicity.

## Materials and methods

The 5-Fluorouracil powder was a kind gift from Pharmedic Laboratories (Pvt.) Ltd. Lahore, Pakistan. MMWCH (190,000–210,000 Dalton, with 75–85% degree of deacetylation), Glutaraldehyde (25% aqueous solution), and Sorbitan monooleate (Span 80) were purchased from Sigma-Aldrich (UK). Dialysis membranes (molecular weight cutoff value 10,000 Daltons) were purchased from Spectrum Labs, Germany. Miglyol oil, acetic acid (99.9% purity), and all other ingredients were of analytical grades.

### Formulation of 5-FU/GA-co-MMWCH-NPs

Glutaraldehyde (GA), based on cross-linked medium molecular weight chitosan nanoparticles were prepared by using the reverse micelles method as described (Radwan et al., 2017). For the aqueous phase, chitosan (MMWCH) was weighed in different amounts and dissolved in 1% v/v acetic acid. The aqueous phase was prepared approximately 5 mg of the drug per 100 mg of polymer solution, mixed, and heated for 2–4 min at 60 °C. For the oil phase, the primary emulsion w/o was prepared using 2 ml of Migylol oil as the external/continuous phase in a 10 ml glass vial using a high-speed homogenizer. Span 80 was used as a surfactant at various concentrations. Both aqueous and oil phase dispersions were stirred with a magnetic stirrer of 1 cm at a different speed for 2–5 h to get a stable emulsion. After homogenization formation of the stable emulsion was achieved and the pH of the solution adjusted to 4–5, various concentrations of cross-linker glutaraldehyde (GA) were added at different time intervals. This dispersion was stirred at a constant stirring speed of 900 rpm at 45 °C for at least 5 h or left overnight with the help of a magnetic stirrer. This dispersion was then centrifuged at 14,000 rpm at 4 °C for at least 15–30 min. The supernatant was decanted, which contains 5-FU-loaded-NPs and pellets were suspended in ethanol for washing off the oil phase. The washing/centrifugation steps were repeated 2–3 times followed by twice washing with methanol and then once with distilled water. Finally, NPs pellets were collected by filtration and lyophilized for further physicochemical description.

### Particle size, polydispersity index (PDI) and zeta potential (mV)

Particle size, PDI, and zeta potential were measured in a Malvern Nano ZS 90 Zeta sizer instrument, the UK, at 25 °C (Gamelin et al., 1996) (24) .

### Drug loading capacity (%LC) and entrapment efficiency (%EE)

The prepared 5-FU loaded NPs formulation is centrifuged at 15,000 rpm for 15 min for settling-down of NPs fragments and quantification of the un-entrapped amount of drug (Zhu et al., 2009; Li et al., 2012) was in the supernatant by using a standard calibration curve of 5-FU via ultraviolet spectroscopy at wavelength 260 nm in triplicate. It was estimated by the following formulas:

$$\%\mathrm{LC}=\frac{\text{Total amount of }5-\text{FU in NPs}-\text{Amount of unentrapped }5-\mathrm{FU}}{\text{Total weight of total NPs}}\times 100$$

$$\text{\%EE }=\frac{\text{Total amount of encapsulated }5-\text{FU in NPs}-\text{Amount of unentrapped }5-\mathrm{FU}}{\text{Total weight of the }5-\text{FU in NPs}}\times 100$$

### Transmission electron microscopy (TEM)

Transmission electron microscopy (TEM-JSM-7500F; JEOL, Tokyo, Japan) is used to determine the morphology of NPs. A tiny drop of aqueous dispersed NPs with adding up of 10 µl of 1% phosphotungstic acid was drop-cast onto a carbon-coated copper grid. This was followed by air drying of the wet grid under strict sink conditions. The grid was loaded on top of the sample holder and inserted in TEM and then photographs of the sample were noted at a suitable voltage and different magnifications power at 10 KeV (Zhu et al., 2009; Liu et al., 2014).

### FTIR spectroscopy (Fourier transform infrared spectroscopy)

FTIR is an analytical technique is used to evaluate interaction and compatibility by identification of functional groups present in NPs formulation components, which determined the stability of the prepared and optimized nanoparticles formulations. FTIR vibrational frequency peaks analysis assembly, that is, Thermo Nicolet spectrometer (Tensor 27 series, Germany), by using KBr pellet method in the range 4000–400 cm^−1^. The empty cell was scanned and taken before sample analysis after locking the pressure arm by putting a small amount of sample on crystal surface KBr disk (Zhang, Cheng, et al., 2008).

### Thermo gravimetric analysis (TGA)

TGA has verified percent weight loss by elevating temperature through the graph between temperature (°C) and percent weight loss done by TA instruments Q600 series Thermal Analysis System (TA instruments, West Sussex, UK). 5 mg of NPs sample weighed and placed in an open pan (platinum 100 µl) attached to a microbalance. The Nps samples were heated at 20 °C/min from 25–500 °C under dry nitrogen at standard mode with ramp test type. NPs sampling was performed in triplicates (Zhang, Cheng, et al., 2008).

### Powder X-ray diffraction studies (PXRD)

X-ray diffractograms by means of a PXRD diffractometer (Bruker D8 Advance, Germany) via Ni-filtered Cu K ά radiation through scanning speed of 0.05°/min having 45 kV source tube voltage, 40 mA electric current and over 10°–60° range of diffraction angle(2*θ*) range (Fournier et al., 2004).

### Drug release studies by UV-Vis spectrophotometer

Drug release behavior of all NPs formulations was done by dissolution studies, which were used as a substitute for *in vivo* performance of 5-FU. A cellulose ester dialysis membrane used by USP dissolution apparatus type II with paddle assembly (Pharma Test, Germany). The dialysis bag (molecular weight cutoff values 10,000 Daltons) was selected on the basis of molecular weight of 5-FU and the size of NPs. Nanoparticles equivalent to 5 mg of 5-FU were taken in a dialysis bag and tied at both ends with thread or clips. The packed dialysis bags were positioned in a vessel of dissolution apparatus containing 250 ml dissolution medium, in both 0.1 N HCl of pH 1.2 and phosphate buffer saline (PBS) of pH 7.4. Dissolution studies were conducted by adjusting the paddle’s speed at 50 rpm and the temperature of media was maintained at 37 °C ± 2 to evaluate pH and thermoresponsive acidic hydrophilic drug release studies (Mukhopadhyay et al., 2015; Radwan et al., 2017). The NPs samples were taken out at predetermined time intervals and their absorbance was taken by UV-Vis spectrophotometer (IRMECO, U2020, Germany), at a *λ*_max_ of 260 nm after 10 ml dilutions. Absorbance was fitted to the straight-line equation of the standard calibration curve to calculate the amount of 5-FU being released from prepared NPs.

### Dissolution kinetics studies

The drug release mechanism from NPs was estimated by the model-dependent approach recommended by the FDA, for comparison of drug release. Model-dependent drug release data were analyzed via various kinetic models, zero-order, first-order, Higuchi, Hixson–Crowell and Korsmeyer–Peppas with DDSolver, an extension of MS. Excel (Mukhopadhyay et al., 2015).

### Physical stability studies

NPs (5 mg/ml), it was noted that %EE of 5-FU MMWCH-NPs stored at, 25 °C, 45 °C, and 4 °C were the most stable formulations in terms of drug retention as the data indicated an insignificant change (*p* > .05) in the %EE of all formulations.

### Acute oral toxicity evaluation

Acute toxicity study protocols were approved by the Pharmacy Research Ethics Committee (PREC), the Islamia University of Bahawalpur, Pakistan (Ref. No. 29-2017-PREC). Albino rabbits of mixed-breed weighing (2.0–2.5 kg) were placed in the animal house of the Department of Pharmacy, the Islamia University of Bahawalpur, for 14 days animals were kept in clean cages light, and darkroom. The animal Laboratory care (Washington DC Press, 2006) was firmly on a typical laboratory diet and common tap water. The rabbits were caged in a well temperature maintained room (at 25 ± 1 °C) and diet as per study protocols, that is, water *ad libitum* was given prior to starting the study. Total six albino rabbits of mixed-breed were randomly divided into three one control group and two treated groups. Each group was fasted for 10 h and was appropriately mark for recognition and holed in wooden boxes throughout the feeding and sampling procedure. Control group rabbits were administered only food and water test or treated group was orally given 30 g/kg and 50 g/kg body weight of dose (optimized NPs and estimated daily intake of food and with glucose water for their survival. All groups were under observations once a day for mortality rate, physical alterations in the skin, fur, eyes, mucus membranes, behavior pattern, tremors, salivation, diarrhea, sleep, and coma for 14 days (Lamiere et al, 2005) Determination of body weights (BW), consumption of food and water were calculated on 0, 1, 7, 14 days of treatment and compared with the control group. According to OECD guidelines, blood samples were drawn from the jugular vein of rabbits from control and treated groups and converted into EDTA tubes for hematological and biochemical analysis. On the 15th-day all rabbits were decapitated for their vital organs, lung, heart, liver, stomach, spleen, gastrointestinal tract (GIT), kidneys, and urinary bladder observations for any abnormality then carefully dissected out for histopathological analysis and weighed (absolute weight) of the vital organs. The relative organ weight (ROW) was calculated by the following equation (Jain et al., 2014):

$$\mathrm{ROW}=\frac{\text{Absolute organ weight }(g)}{\text{Body wt}.\text{ of rabbit on sacrifice day}}\times 100$$

### Statistical analysis

Student’s *t*-test was employed for comparison of data (*n* = 3) and *p* ≤ .05 was considered as statistically significant to the estimation of the effect of variable volumes of cross-linker (GA) on %LC, %EE, particle size, PDI, and Zeta Potential (mV) on optimized NPs formulation.

## Results and discussions

### Effect of GA concentration on particle size distribution and %EE of NPs

Table 1 clearly determines the composition of prepared NPs formations of 5-FU/GA/MMWCH-NPs. Results in Table 2 showed that an increased concentration of cross-linker led to small size NPs from 5-FU1 to 5-FU6 (Ppt to 230 nm) and decreased in zeta potential (mV) from 5-FU1 to 5-FU6 (- to −15.0 mV), while %EE of NPs from 5-FU1 to 5-FU6 was increased (NA to 59%), respectively. Our optimized formulation (5-FU6) has a particle size of 230 nm, 59% of entrapment efficiency (%EE), 1.43% of loading capacity (%LC), zeta potential (mV) −15.01 mV and polydispersity index (PDI) was decreased to 0.15, respectively. This is due to highly cross-linked Nanoparticles, indicating a strong interaction through covalent bonds between chains of polymer (MMWCH) and functional cross-linking agent (GA). Optimized formulation (5-FU6) was formulated, designed and developed which has all the necessary standards to be manufactured on a large scale for the incorporation of the active drug for good cancerous patient’s compliance (Zhang, Shen, et al., 2008; Bagre et al., 2013).

**Table 1.** Formulation composition of 5-FU/GA-MMWCH-NPs.

Formulation code	5-FU % (w/v)	MMW CH% (w/v)	Migylol oil% (w/v)	Span 80% (w/v)	GA % (w/v)
5-FU1	5 mg	0.50	02	01	0.25
5-FU2	5 mg	0.60	02	01	0.25
5-FU3	5 mg	0.70	02	01	0.25
5-FU4	5 mg	0.80	02	01	0.25
5-FU5	5 mg	0.90	02	01	0.25
5-FU6	5 mg	0.10	02	01	0.25

**Table 2.** Influence of GA concentrations on particle diameter and %EE of NPs.

Formulation code	GA % (w/v)	Mean size (nm)	PDI	ZP (mV)	EE (%)	LC (%)
5-FU1	0.05	Ppt.	NA	−18.0	NA	NA
5-FU2	0.10	350	0.11	−20.1	69	1.70
5-FU3	0.15	300	0.14	−11.0	59	1.42
5-FU4	0.20	285	0.17	−12.0	39	1.48
5-FU5	0.25	285	0.17	−20.5	40	1.50
5-FU6	0.3	230	0.18	−15.0	59	1.43

### Effect of stirring speed on particle size distribution and %EE of NPs

From results in Table 3 showed that an increase in stirring speed leads to small size NPs from 5-FU1 to 5-FU6 (380–230 nm) and decreased in zeta potential (mV) from 5-FU1 to 5-FU6 (−18.0 to −15.0 mV, while %EE of NPs from 5-FU1 to 5-FU6 was increased (10 to 59%), respectively. Our optimized formulation (5-FU6) has a particle size of 230 nm, 59% of entrapment efficiency (%EE), zeta potential (mV) −15.01 mV, and polydispersity index (PDI) was decreased to 0.15, respectively. Reduction in size of optimized formulation 5-FU6 seems an increase in surface area of particles indicating fast release of a drug but due to cross-linking agent, it remained in a controlled fashion (Li et al., 2012; Mukhopadhyay et al., 2015).

**Table 3.** Influence of stirring speed on particle diameter & %EE of NPs.

Formulation code	Stirr-ing rate rpm	Mean size (nm)	PDI	ZP (mV)	EE (%)	LC (%)
5-FU1	300	380	0.15	−18.0	10.	NA
5-FU2	400	350	0.16	−20.1	12.	1.70
5-FU3	500	300	0.16	−11.0	24.	1.43
5-FU4	600	285	0.16	−12.0	32.	1.48
5-FU5	700	285	0.16	−20.5	48.	1.50
5-FU6	900	230	0.15	−15.0	59	1.43

### Effect of stirring time on particle size distribution and %EE of NPs

**Figure 1.**
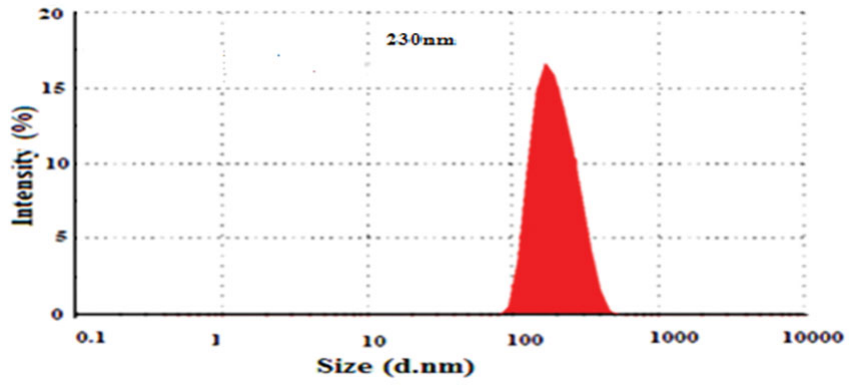
Mean particle diameter of 5-FU6.

**Figure 2.**
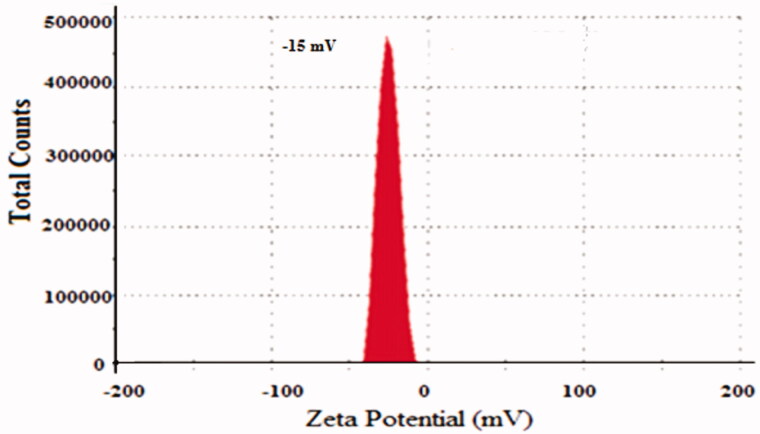
Zeta Potential of 5-FU6.

Results in Table 4 showed that an increased stirring time leads to small size NPs from 5-FU1 to 5-FU6 (350–230 nm) and decreased in zeta potential (mV) from 5-FU1 to 5-FU6 (−19.0 to −15.0 mV, while %EE of NPs from 5-FU1 to 5-FU6 was increased (0 to 58.9%), respectively. Our optimized formulation (5-FU6) has a particle size of 230 nm, 58.9% of entrapment efficiency (%EE), zeta potential (mV) −15.01 mV as shown in Figures 1 and 2. A sharp decrease in particle size was demonstrated with increased stirring time; it would be due to decreased interaction parameters between polyelectrolyte and solvent (Fournier et al., 2004). Our results were in agreement with the data that has been already published by Radwan and co-researchers as they evaluated that with increased stirring time, acceleration of liquid dispersion occurred as it found to be increased electric forces between them as well as narrow dispersity index (PDI) that decreased size of MMWCH-NPs while increased % EE of NPs (Fournier et al., 2004; Zhang, Cheng, et al., 2008; Radwan et al., 2017).

**Table 4.** Influence of stirring time on particle diameter & %EE of NPs.

Formulation code	Stirr-ing time (min)	Mean size (nm)	PDI	ZP (mV)	EE (%)	LC (%)
5-FU1	30	380	0.19	−18.0	NA	NA
5-FU2	40	350	0.18	−20.1	12.10	1.70
5-FU3	50	300	0.16	−15.1	24.90	1.42
5-FU4	60	285	0.17	−12.0	32.00	1.48
5-FU5	70	285	0.18	−20.5	48.50	1.50
5-FU6	80	230	0.15	−15.0	58.90	1.43

**Table 5.** Stability studies of 5-FU6 for 6 months as per particle diameter (nm) and drug contents.

Months	Room temperature	45 °C	4 °C
Particle size (nm)			
0	300	300	300
1	300	300	300
2	300	300	299
3	399	399	398
4	398	398	398
5	399	399	398
6	399	398	398
Drug contents			
0	100	100	100
1	98.5	98	98
2	97.5	97	97
3	98	98	99
4	98	98	98
5	98	99	98
6	98	98	98

### Drug loading and entrapment efficiency (%LC & %EE)

%LC and %EE of 5-FU loaded MMWCH-NPs were measured by the indirect method as it showed a positive correlation between LC% & %EE, as it was found to be in Table 2 for 5-FU1–5-FU6, (%LC 0.03–0.22%), while (%EE 12–59%), respectively. The formulation which was optimized 5-FU6, noted %LC 0.22, with greater Entrapment efficiency 59%, it might be due to less number of stable binding with cross-linker glutaraldehyde (Gupta and Jabrail, 2007). Our results are in accordance to previously reported that with increased concentration of polymer, cross-linking agent, speed and time of cross-linking loading capacity (%LC) and entrapment efficiency (%EE) will decrease due to hindrance in the homogenous distribution of crosslinker, leading in large size particles (Mukhopadhyay et al., 2015).

### Fourier transmission infrared spectroscopy (FTIR)

**Figure 3.**
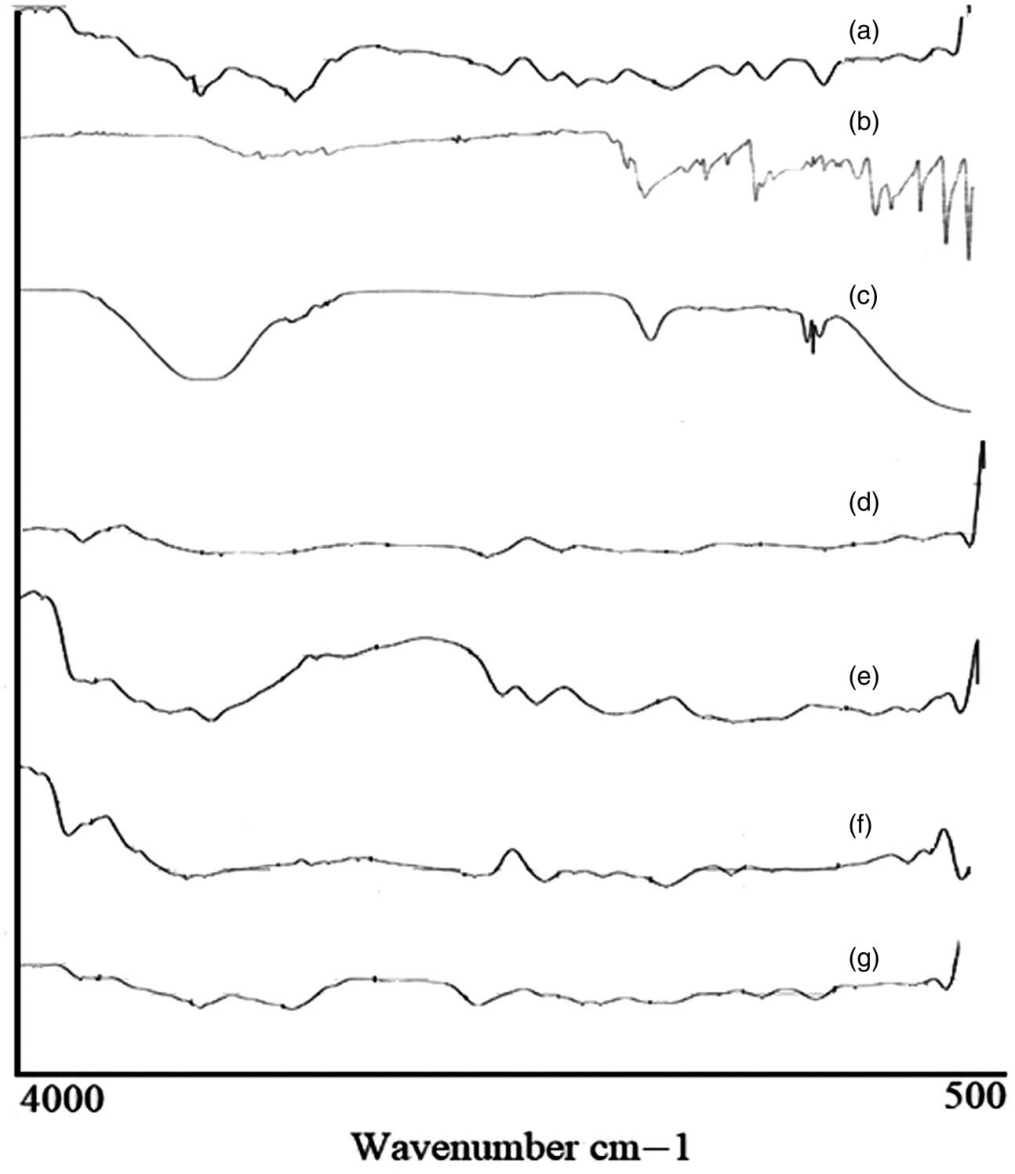
FTIR Spectrum of (a) pure MMWCH, (b) Migylol oil, (c) GA, (d) Span 80, (e) unloaded MMWCH-NPs, (f) pure 5-FU, (g) Loaded MMWCH-NPs.

FTIR spectra of pure medium molecular weight chitosan (MMWCH), migylol oil, glutaraldehyde (25% aqueous solution), span 80, unloaded medium molecular weight cross-linked chitosan nanoparticles, pure 5-FU, loaded medium molecular weight chitosan (5-FU6) are given in Figure 3(a–g). In FTIR spectra of pure medium molecular weight chitosan (MMWCH), it showed a characteristic peak at 3596 cm^−1^ depicted stretching vibration of –OH group and symmetrical N–H group stretching vibration (Mukhopadhyay et al., 2015). The symmetric stretching band peak of –CH_2_ group at 2872.74 cm^−1^ was observed which corresponded to C-6 of the pyranose group (Zhang, Cheng, et al., 2008). The characteristics band peak at 1664.41 cm^−1^ described C = O of acetamide group and high-intensity band peak at 1526 cm^−1^ represented –NH bending vibration of NH_2_ group (Mukhopadhyay et al., 2015). The stretching vibration band peak at 1048.28 cm^−1^ illustrated (C–OH groups (Jain et al., 2014) respectively, shown in Figure 3(a). FTIR Sectral of Migylol oil was demonstrated a characteristic bend peak of the O–H group at 3500.75 cm^−1^ and a sharp absorbance peak pattern at 2987.66 cm^−1^ indicative of the N–H stretching of the primary amino group respectively (Liu et al., 2014). The stretching band peak of the C–O group at 1035.77 cm^−1^, an intense band peak of C–H group at 1732.5 cm^−1^ and N–H group bending at 1532.41 cm^−1^ respectively (Zhu et al., 2009) as shown in Figure 3(b). FTIR spectra of glutaraldehyde (25% aqueous solution), showed the broadband peak from 3000 to 3400 cm^−1^ which was attributed to the stretching vibration of the O–H and N–H groups respectively. It was associated with the previously reported hydrogen-bonded molecule (Li et al., 2012). While it displayed a very weak peak at 1690 cm^−1^, which was associated with Schiff base of group C=N and at 1580 cm^−1^ which was C=C stretching vibration peak respectively, which confirmed the aldol condensation of oligomer (Mukhopadhyay et al., 2015). The band peak at 1700 cm^−1^ was the confirmation of the vibration peak of the aldehyde group while the specific band stretching of amide I (C=O) band at 1690 cm^−1^ and amide II (–NH) at 1589 cm^−1^, respectively as was evident in Figure 3(c). FTIR Spectra of pure emulsifying agent sorbitan monooleate (Span 80), showed various intense characteristic peaks pattern, the band peak at around 3120–3601 cm^−1^, is demonstrated to –NH_2_ and –OH stretching vibration, associated with the molecular hydrogen bonding of the molecule. The characteristics absorption band appeared at 1750 cm^−1^, of amide I (C=O) band and amide II (–NH) at 1447 cm^−1^, amide III band peak at 1085 cm^−1^ respectively, while the spectra were normalized by the absorbance of the asymmetric stretching of C–O at around 729 cm^−1^ as illustrated in Figure 3(d). FTIR spectra of unloaded medium molecular weight chitosan nanoparticles (GA-co-MMWCH -NPs); demonstrated prominent peaks pattern at 3297.5 cm^−1^ indicative of (N-H) group stretching of the primary amino group, at 1158.7 cm^−1^ was illustrated stretching band peak of (C–O) group, band peak of (C–H) at 1425 cm^−1^, bending peak of (N–H) group at 1506 cm^−1^, O-H group at 3359 cm^−1^ respectively, while, after chemical cross-linking, the characteristic sharp peak at 1650 cm^−1^ of bond C=N was shifted to 1630 cm^−1^ and at 1506 cm^−1^ stable imine group (–NH) of Schiff base was formed due to strong interaction of medium molecular weight chitosan (MMWCH), and cross-linking agent glutaraldehyde (GA), even though nanoparticles were washed with ethanol and water to remove the excess volume of glutaraldehyde, band peak at 1711 cm*^−^*^1^ indicated non-reacted aldehyde group of glutaraldehyde (Jain et al., 2014) as it is evident in Figure 3(e). FTIR spectra of the pure drug (5-FU), absorption band peaks, in the region between 3000-2900 cm^−1^ correspond C‒H stretching. A broadband spectral peak of N–H stretching vibrations (Fournier et al., 2004) was noted at 2930 cm^−1^ of C-H stretching vibrations. Characteristics band peaks at 2390.71 cm^−1^ and 2342.01 cm^−1^ of ‒CH_2_‒ asymmetric vibrations. Bending peaks at 1731.31 cm^−1^ for C=O stretching, C–N stretching at 1598.16 cm^−1^, respectively. C‒H stretching peak in-plane at1242.71 cm^−1^ and for C–F stretching at 1255.20 cm^−1^ and for pyrimidine ring vibration at 752.53 cm^−1^, respectively (Zhang, Cheng, et al., 2008) as shown in Figure 3(f). In FTIR Spectra of 5-FU-loaded cross-linked medium molecular weight chitosan NPs and when 5-FU was incorporated into unloaded (GA/MMWCH-NPs) many characteristics peak in its FTIR were disappeared which is confirmation of strong interaction between 5-FU and medium molecular weight chitosan when 5-FU is being entrapped in GA/MMWCH-NPs, a characteristics absorbance peak of 5-FU at 1665 cm^−1^ which corresponding to (C=O) vibration, shifted to 1638 cm^−1^ and overlapped to the amino group of NPs. This FTIR spectrum is determined by the presence of 5-FU into the unloaded medium molecular weight crosslinked chitosan nanoparticles. Lie and his colleagues have already been published their studies; the FTIR peak fashion was the confirmation of the strong interaction between pure chitosan, medium molecular weight chitosan (MMWCH) matrix with that of 5-FU and these characteristics intense peaks manner demonstrated that 5-FU exists successfully inside the core of nanoparticles (NPs) in amorphous or solid solution form so such data results are similar findings have been published (Mukhopadhyay et al., 2015). Hence our findings are in line with the previous researches as shown in Figure 3(g). Therefore, FTIR spectra were used to analyze physicochemical, chemical interaction due to cross-linker glutaraldehyde (GA), between pure drug (5-FU), pure polymer (MMWCH) in all synthesized formulations, and an optimized formulation (5-FU6) to make sure that there is no incompatibility variability present in between drug and formulation components, on the whole, all formulations 5-FU1 to 5-FU6 are stable due to the formation of stabilized colloidal dispersion. In physicochemical interaction splitting of absorption, peaks were shown while in chemical interaction some prominent peaks were disappeared during our study.

### Powder X-ray diffraction pattern (PXRD)

**Figure 4.**
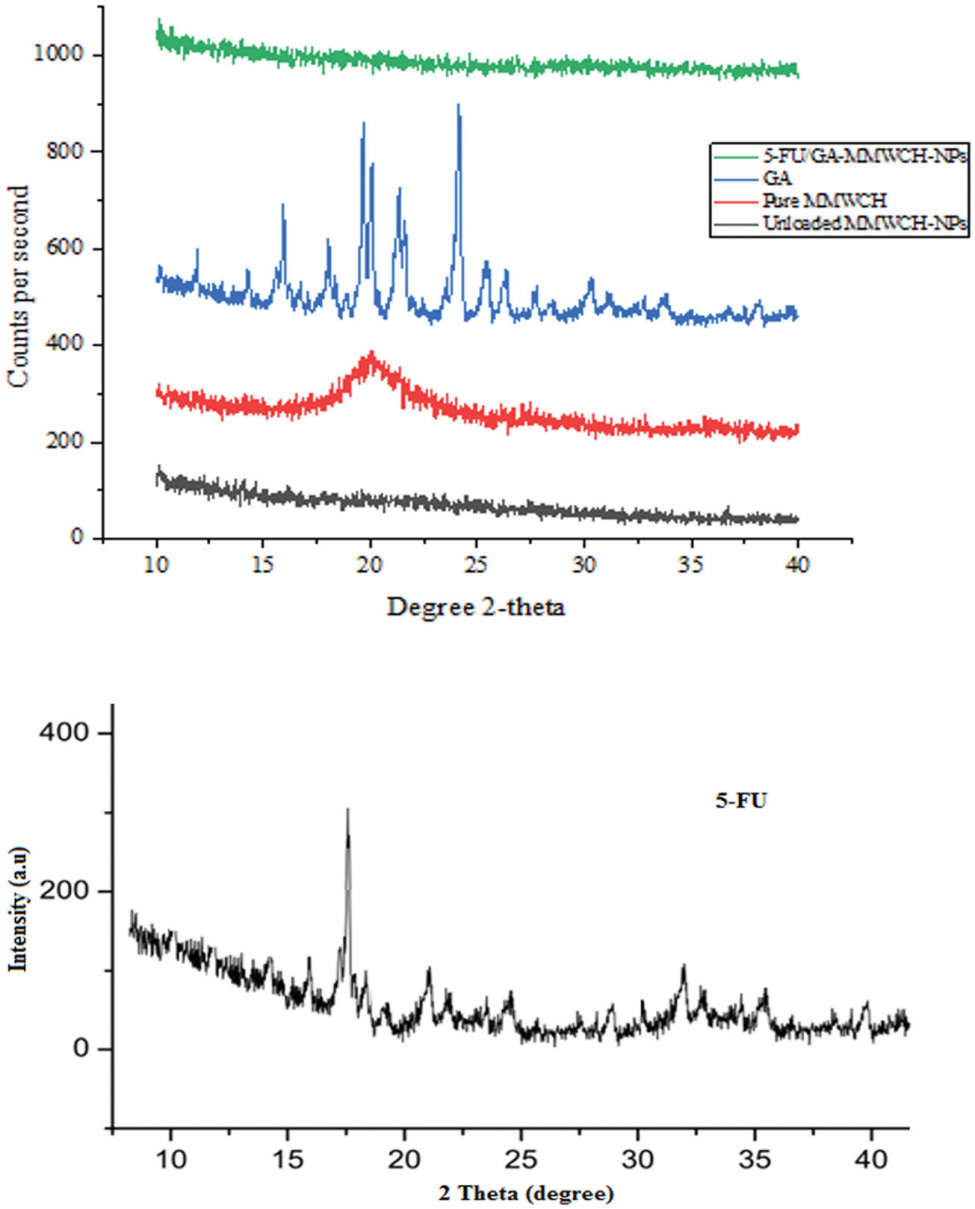
(A–E) PXRD of 5-FU/GA-MMWCH-NPs, GA, pure MMWCH, unloaded MMWCH-NPs, and PXRD diffractogram of pure 5-FU.

The discrete and intense peaks of pure medium molecular weight chitosan (MMWCH), cross-linker glutaraldehyde (GA), GA/MMWCH-NPs (unloaded NPs), 5-FU, and 5-FU-GA/MMWCH-NPs (loaded NPs) were established which demonstrated their crystalline nature with insignificant amorphous contents are shown in Figure 4(A–E). PXRD diffraction pattern of the polymer of medium molecular weight chitosan (MMWCH) was shown moderately sharp diffraction at 2*θ* of 12.5°, 15.2°, 18.5°, 20.1°, 23.5°, and 31.5°, and less diffraction at 2 *θ* of 22.5°, 34.6°, 35.8° and 39.5° (Mukhopadhyay et al., 2015), respectively as shown in Figure 4(A). PXRD diffraction pattern of cross-linker glutaraldehyde (25% aqueous solution) was displayed no intense characteristic peaks, only hump peaks patterns were observed (Fournier et al., 2004; Jain et al., 2014; Mukhopadhyay et al., 2015) as illustrated in Figure 4(B).

PXRD pattern of cross-linked medium molecular weight chitosan nanoparticle (GA/MMWCH-NPs) showed less crystallinity than pure chitosan as polymers usually amorphous or semi-crystalline in their nature. Drop off effect in crystallinity might be occurred due to the incorporation of the heavier group within chitosan because of cross-linker glutaraldehyde (Zhang, Cheng et al., 2008). PXRD spectra of cross-linked medium molecular weight chitosan NPs with cross-linker glutaraldehyde (GA) which showed two broad peaks at 2*θ* = 12° and 2*θ* = 32°, indicates from crystalline to amorphous nature of MMWCH. Also, the shift in 2*θ* values confirmed effective cross-linking (Yuan et al., 2007), as it is well known that rigidity of the crystalline structure of MMWCH is stabilized by intra and intermolecular linkages between the aldehyde group (Jain et al., 2014) of cross-linker and the amino group of polymer MMWCH, as shown in Figure 4(C). PXRD diffractogram of 5-Fluorouracil had shown a single sharp peak and the highest one at 2*θ* equals 16.1° determines crystalline nature of 5-FU, while some moderate intense peaks at 15.1°, 19.5°, 24.06°, 30.35°, and 35.49° at 2*θ* scale (Zhang, Cheng, et al., 2008; Jain et al., 2014), corresponding to crystalline nature of 5-Fluorouracil as shown in Figure 4(D). In PXRD of loaded NPs, it has been reported that gradual disappearance of crystalline nature as well as amorphous character of loaded (5-FU/GA-MMWCH-NPs) occurred with an increase in 2*θ* scale. However, all other 5-FU/GA-MMWCH-NPs formulations, which confirmed the transformation of 5-Fluorouracil into amorphous nature or solid solution form due to internment of the pore and entanglement of the amino group of polymer meshwork with aldehyde group of the cross-linking agent as shown in Figure 4(E). Similarly, previous data proved that other peaks vanished when 5-FU was loaded with MMWCH and most peaks were hump (Li et al., 2012). In our current research work, similar results were observed. Hence, current study findings confirmed the results of the previous studies.

### Transmission electron microscopy (TEM)

**Figure 5.**
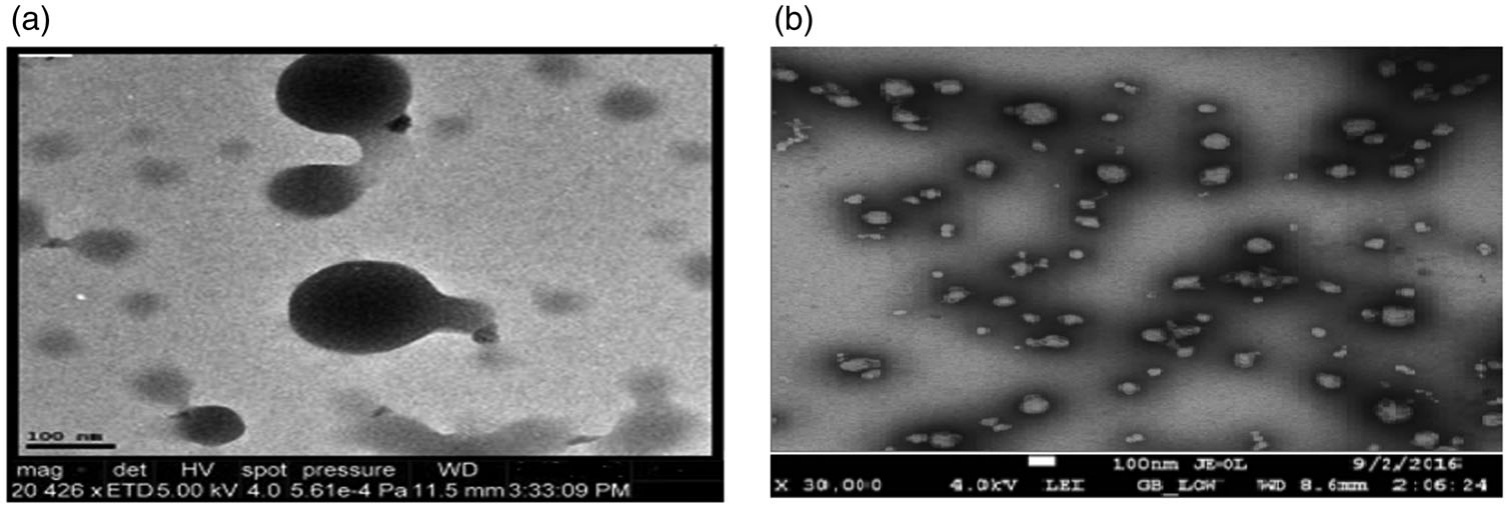
TEM micrograph of (a) unloaded MMWCH-NPs, (b) loaded 5-FU/GA-MMWCH-NPs.

The shape and internal morphology of unloaded medium molecular weight cross-linked chitosan nanoparticles (GA-MMWCH-NPs) and 5-FU loaded medium molecular weight chitosan nanoparticles (5-FU/GA-MMWCH-NPs), are shown in Figure 5(a,b). Figure 5(a), as shown TEM images of unloaded MMWCH-NPs which were spherical in shape and fairly smooth surface without aggregation, while the average diameter is found to be 200–350 nm with monodispersity index of NPs (Li et al., 2012), in Figure 5(b), as shown TEM images of loaded medium molecular weight chitosan nanoparticles (5-FU/GA-MMWCH-NPs), which were spherical in shape and homogeneous surface with small aggregation nanosized while the average diameter is found to be 150–250 nm with polydispersity index of Nanoparticles (Zhang, Shen, et al., 2008; Wang et al., 2017). The smooth surface of nanoparticles is recognized as the maximum encapsulation of drug as well chemical cross-linking during the development process of nanoparticles (Glavas-Dodov et al., 2013; Pratt et al., 2013). Our current study demonstrated the stable colloidal dispersion of Nanoparticles which are further applicable for pharmacokinetic studies that might be a prerequisite its potential use to enhance the bioavailability of 5-Fluorouracil by reducing toxicity.

### Thermal gravimetric analysis (TGA)

**Figure 6.**
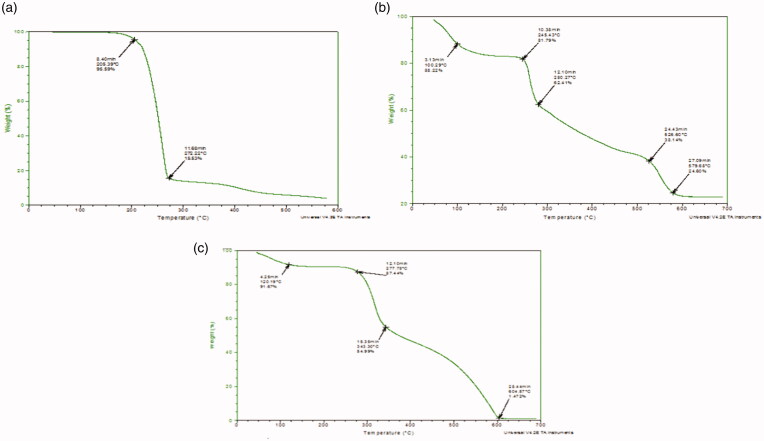
TGA graph of (a) pure 5-FU, (b) PM (5-FU/GA-MMWCH-NPs), (c) 5-FU6-NPs (5-FU/GA-MMWCH-NPs).

The TGA graph determined the percentage of weight loss (Weight (%) of the material during the heating phase. The Thermal gravimetric curve of 5-FU, Physical mixture of loaded NPs (5-FU/GA/MMWCH-NPs), and optimized formulation (5-FU6-NPs) are given in Figure 6(a–c). In TGA curve of 5-FU, thermogravimetric analyzer demonstrated an initial weight loss of 4.41% at 205.39 °C (Hong et al., 2007; Pratt et al., 2013) which was actually a loss in moisture contents and it was showed an abrupt loss of weight which was due to the phase transition temperature of 5-FU and it was detected of 80.06% at 272.22 °C (Shu & Zhu, 2001; Li et al., 2012) as it shown in Figure 6(a). In TGA curve of physical mixture of 5-FU loaded medium molecular weight chitosan NPs (PM). The thermogravimetric analyzer was established an initial weight loss of 11.72% at 100.29 °C, which was a loss in moisture while during the second range weight loss was of 6.43% at 245.43 °C. Third loss of moisture contents of 24% at 526.60 °C, while terminal weight loss of 13.81% at 979.68 °C (Hong et al., 2007), corresponding to the complex process including dehydration of the saccharide rings, decomposition of acetylated deacetylated units of medium molecular weight chitosan and cross-linking of glutaraldehyde. The maximum peak of the physical mixture was noted at 245.43 which was in fact phase transition temperature of the drug peak (Shu & Zhu, 2001; Kildeeva et al., 2009), as shown in Figure 6(b). In TGA curve of optimized nanoparticle formulation containing 5-FU loaded CH-NPs (5-FU6-NPs)., which was established an initial weight loss at 120.19 °C, is due to evaporation of water contents while during second range (277.78–604.57 °C) (Zhang, Shen, et al., 2008; Wang et al., 2017) corresponded to cross-linking of glutaraldehyde (GA). The maximum peak of developed NPs formulation was noted at 277.78 °C (Michael et al., 2007; Patel et al., 2014), slight shifting of the phase transition temperature of 5-FU peak, reveals to efficient entrapment of 5-FU within NPs sample and indicates the maximum thermo-stability of NPs, as shown in Figure 6(c).

### Acute toxicity studies

Acute toxicity study protocols were approved by the Pharmacy Research Ethics Committee (PREC), the Islamia University of Bahawalpur, Pakistan. The rabbits were caged in a well temperature maintained room (at 25 ± 1 °C) and diet as per study protocols, that is, water *ad libitum* was given prior to starting the study. Total eighty albino rabbits of the mixed breed were randomly divided into three groups (group I control, group II, & group III test). Each group was fasted for 10 h and was appropriately mark for recognition and holed in wooden boxes throughout the feeding and sampling procedure. Control group rabbits were administered only food and water test or treated groups were orally given 30 mg/kg & 50 mg/kg body weight of dose (optimized NPs) estimated daily intake of food and with glucose water for their survival. All groups were under observations once a day for mortality rate, physical changes in the skin, fur, eyes, and mucus membranes, behavior pattern, tremors, salivation, diarrhea, sleep, coma, body weights (BW), consumption of food and water, hematological, biochemical, histopathological analysis for 14 days (George & Shipp, 2000; Halim et al., 2011).

### Stability studies

From the results as shown in Table 5, optimized formulation was selected and stability studies were carried out for this selected optimized formulation. Variation in particle size and drug content was not significant before and after stability studies. 5-FU6-NPs formulation was estimated for stability at room temperature (25 °C ± 75% relative humidity) for 6 months. The optimized 5-FU6 formulation was found to be stable overstudied stability period and no significant changes in FTIR and PXRD were seen. The findings of the stability studies (‘0’ day to 6 months) were statistically analyzed by applying *t*-test and it was found that these results were insignificant (*p* < .05) confirming the stability of synthesized and optimized NPs formulation.

### Drug release studies by UV-Vis spectrophotometer

**Figure 7.**
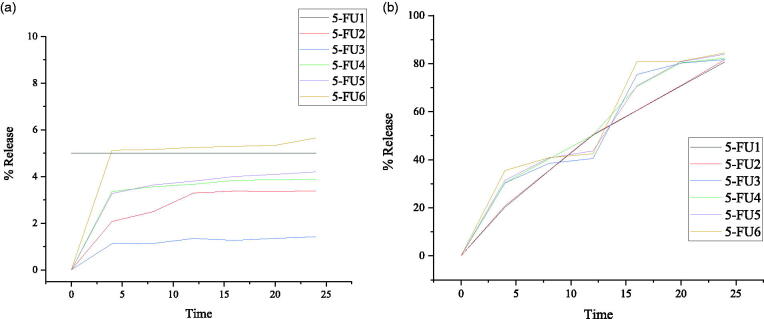
*In vitro* percentage release of (a) 5-FU loaded MMWCH- NPs in buffer pH 1.2, (b) 5-FU loaded MMWCH- NPs in buffer pH 7.4.

Drug release study was carried out for prepared 5-FU loaded medium molecular weight chitosan NPs (prepared with respect of different concentrations of medium molecular weight chitosan, different concentrations of crosslinker (GA) at a different temperature, times, and rotation speeds, respectively). Liberation of 5-Fluorouracil from medium molecular weight chitosan NPs depends on the extent of cross-linking, chemical reaction, and solvent compatibility. In Table 6, cumulative *in-vitro* drug release of NPs formulations 5-FU1–5-FU6 during 24 h period of incubation at 37 °C with the corresponding plot in Figure 7(a,b) at both low and high pH (pH 1.2 & pH 7.4. Therefore, an insignificant drug release data was observed at pH 1.2 while at pH 7.4 after the 4th-hour, initial cumulative drug release % age from FU1 to 5-FU6 was 20.21–35.40%, at the 8th-hour, drug release from 5-FU1 to 5-FU6 was 35.40–40.85%, at 12 h, drug release from 5-FU1 to 5-FU6 was 42.50–50.21%, at 16th-hour, drug release from 5-FU1 to 5-FU6 was 60.50–80.87%, at 20th-hour, drug release from 5-FU1 to 5-FU6 was 5-FU3 was 70.70–80.98%, at the end of 24th-hour drug release from 5-FU1 to 5-FU6 was 80.74–84.46%. Overall, the release of 5-fluorouracil with respect to time was 0–84.46% from 5-FU1 to 5-FU6, respectively. When the concentration of polymer (MMWCH) was increased, polymer viscosity leading to a less diffuse meshwork which hinders slow drug release from NPS’s core (Zhang, Cheng, et al., 2008). When the volume of glutaraldehyde (GA) was increased, drug release decreased; due to highly stable cross-linked MMWCH-NPs, with high density as well as reduced drug diffusion process (Wang et al., 2017). An increase in stirring speed beyond 900 rpm has reduced particle size thus increased surface area in contact with dissolution medium, resulting in faster drug release but due to cross-linker drug release found to be in controlled fashion (Patel et al., 2014). An increase in time of cross-linking up to 90 min favored the controlled release of drug from cross-linked MMWCH-NPs. This is because of the hardening of cross-linked MMWCH-NPs, resulting in sustained/prolong drug release from cross-linked MMWCH-NPs (Patel et al., 2014).

**Table 6.** *In vitro* % age release of 5-FU from MMWCH-NPs in buffer pH 7.4 at 37 °C.

Time (h)	5-FU1 (%)	5-FU2 (%)	5-FU3 (%)	5-FU4 (%)	5-FU5 (%)	5-FU6 (%)
0	0	0	0	0	0	0
4	20.21	20.82	30.31	30.45	31.50	35.50
8	35.40	35.50	38.50	40.34	40.70	40.85
12	50.21	50.52	40.52	50.25	43.70	42.50
16	60.50	60.59	75.51	70.52	70.80	80.87
20	70.70	70.90	80.21	80.35	80.82	80.98
24	80.74	81.70	81.80	82.30	83.90	84.46

The point to be noted that after 24 h incubation the maximum release rate was 84.46% due to the stability of cross-linked MMWCH-NPs in PBS solution. Drug release from NPs is based on diffusion instead of degradation or erosion otherwise it is predictable that 100% release of drug could be obtained in the presence of an enzyme in the colon. Our selected optimized 5-FU6 NPs formulation showed the capability of releasing the drug efficiently in a sustained manner and could possibly be used for further investigation as a therapeutic product in acute oral toxicity studies of anticancer agents.

### Dissolution kinetics studies

Figure 7(a,b) and Table 6 explain cumulative percent of 5-FU released after 24 h dissolution from optimized (5-FU6) NPs formulation showed pH-dependent drug release profile. 5-Fluorouracil release increased gradually on increasing pH of dissolution media. The release of 5-FU was slow down with an increase percentage of cross-linking agent, that is, glutaraldehyde (GA). The sustained release effect was due to cross-linked networking of NPs which delay the rapid release of hydrophilic drug from cross-linked chitosan nanoparticles. Zero-order showed the highest values of the regression coefficient (*R*^2^) instead of the Higuchi model. Therefore, the best fit model followed by the Higuchi model is the Fickian diffusion mechanism of 5-FU release from MMWCH-NPs on this 7.4 pH at 37 °C as shown in Table 7.

**Table 7.** *In vitro* percentage of drug release kinetic data.

Formulations	Zero order		First order		Higuchi		Hixson–Crowell		Kersmeyer–Peppas	
	*R*^2^	*K*_0_	*R*^2^	*K*_1_	*R*^2^	*K*_H_	*R*^2^	*K*_HC_	*R*^2^	*N*
5-FU1	0.190	7.260	0.851	0.321	0.871	12.61	0.885	0.042	0.972	0.326
5-FU2	0.188	6.547	0.892	0.190	0.921	11.18	0.873	0.030	0.971	0.330
5-FU3	0.135	7.345	0.895	0.265	0.905	16.89	0.856	0.041	0.972	0.334
5-FU4	0.991	8.340	0.903	0.165	0.871	17.17	0.884	0.021	0.971	0.328
5-FU5	0.891	9.241	0.931	0.174	0.851	14.45	0.873	0.030	0.977	0.290
5-FU6	0.906	8.654	0.881	0.156	0.966	16.65	0.854	0.031	0.976	0.256

### Acute oral toxicity for developed carriers

Acute oral toxicity of any therapeutic agent is the study of the therapeutic index of therapeutic agents or drugs (LD50/ED50). Developed carrier system 30 mg/kg MMWCH-NPs and 50 mg/kg NPs were implemented with protocols of Organization of Economic Co-operation and Development (OECD) of chemicals toxicity (Canadian Council on Animal Care, 1993a). This developed carrier system demonstrated a broad spectrum of activity to reduce acute toxicity and enhance therapeutic efficacy. There were insignificant variations for all examined tissues during acute toxicity in skin irritation, abnormality in behavior, biochemical and histopathology toxicity. No toxicity was confirmed within 2 weeks of the study. 5-Fluorouracil cross-linked chitosan nanoparticles in acute toxicity study revealed that toxicologic changes associated with body weight, water, feed consumption, hematology, clinical chemistry, and organ weight were not more than 30 mg/kg/day. In 2 weeks acute toxicity study, we observed the absence of mortality even at the highest doses. There were no toxicological changes up to 50 mg/kg/day of CH-NPs (Canadian Council on Animal Care, 1993b; Chao & Zhang, 2012; Chen et al., 2014). Nanoparticles regard body weight, feed and water utilization, eye examination, brain functioning, blood cells etc. Therefore, the no-observable-adverse-effect-level (NOAEL) for tested rabbits was considered to be 50 mg/kg/day (De Campos et al., 2001; Fielden & Kolaja, 2008).

### Clinical examinations

Normal clinical observations were seen in all three groups of rabbits. No harmful affected were observed in all vital organs throughout 2 weeks of treatment as illustrated in Table 8 after oral administration of 5-FU-MMWCH-NPs & Oral solution of 5-FU-GA-co-MMWCH-NPs no-observable-adverse-effect-level (NOAEL), on body weight, nutrients consumption, poisoning complications, no dermal irritation, and behavioral deviation such observations confirmed that our NPs formulations were devoid of clinical toxicity (Kennedy et al., 1986; George & Shipp, 2000). NOAEL, of illness (i.e. vomiting, eye secretion, running nose, and salivation) were confirmed after oral administration of 5-FU loaded cross-linked chitosan NPs. The followings are findings of under observations parameters as listed in Table 8.

**Table 8.** Clinical examinations of optimized NPs formulation and comparison with control.

Clinical observations	Group I (Control)	Group II	Group III
		(5-FU-MMWCH-NPs solution) 30 mg/kg	(5-FU-MMWCH NPs solution) 50 mg/kg
Clinical complaints	Nil	Nil	Nil
Body wt. (kg)			
Prior treatment	1.99	1.96	2.00
1st Day	1.98	1.96	2.00
7th Day	2.02	2.00	2.03
14th Day	2.04	2.04	2.04
Intake of liquid (ml)			
Prior treatment	198	193	202
1st Day	195	198	196
7th Day	203	200	193
14th Day	205	202	200
Intake of food (g)			
Prior treatment	69	68	74
Day 1	73	62	69
Day 7	69	69	79
Day 14	75	70	74
Skin toxicity			
Skin irritation	Nil	Nil	Nil
Ocular toxicity			
Simple pain	Nil	Nil	Nil
Mortality	Nil	Nil	Nil

Mean± (*n* = 3).

### Biochemical blood analysis

Prepared and optimized NPs formulations were given to rabbits and any defect in blood chemistry as blood samples were drawn from rabbits for biochemical blood analysis by a hematology Analyzer. Variables parameters during blood analysis including red blood cell count (RBC), white blood cell count (WBC), hemoglobin (HB), platelet count, mean corpuscular hemoglobin (MCH), mean corpuscular volume (MCV), mean corpuscular hemoglobin concentration (MCHC), hepatic, kidney, & lipid analysis (Olson et al., 2000; ICH Guidance for Industry, 2005; Hureaux et al., 2010) as illustrated in Tables 9 and 10.

**Table 9.** Biochemical/blood analysis.

Clinical hematology	Group I (Control)	Group II	Group III
Hb, g/DL	7	14	14
Ph	7	7	7.1
WBCs, ×10^9^/L	7	7.1	7.2
RBCs, ×10^6^/mm^3^	6	6.3	5.9
Platelets, ×10^9^/L	4	4.05	4.10
Monocytes, %	3	3.0	3.1
Neutrophils, %	50	52	53
Lymphocytes, %	70	67	69
MCV, %	62	63	63.5
MCH, pg/cell	22	21.2	21.8
MCHC, %	31	30.8	31.3

**Table 10.** Profile of liver, kidney and lipid.

Biochemical	Group I	Group II	Group III
ALT(IU/L)	150	160	165
AST (IU/L)	65	75	80
Creatinine(mg/dL)	1.28	1.30	1.30
Urea(mmol/L)	14.5	15.5	15.5
Uricacid(mg/dL)	03	2.8	03
Cholesterol(mg/dL)	72	78	72
Triglyceride(mmol/L)	01	1.5	1.3

Mean ± (*n* = 3).

### Histology and pathology analysis

**Figure 8.**
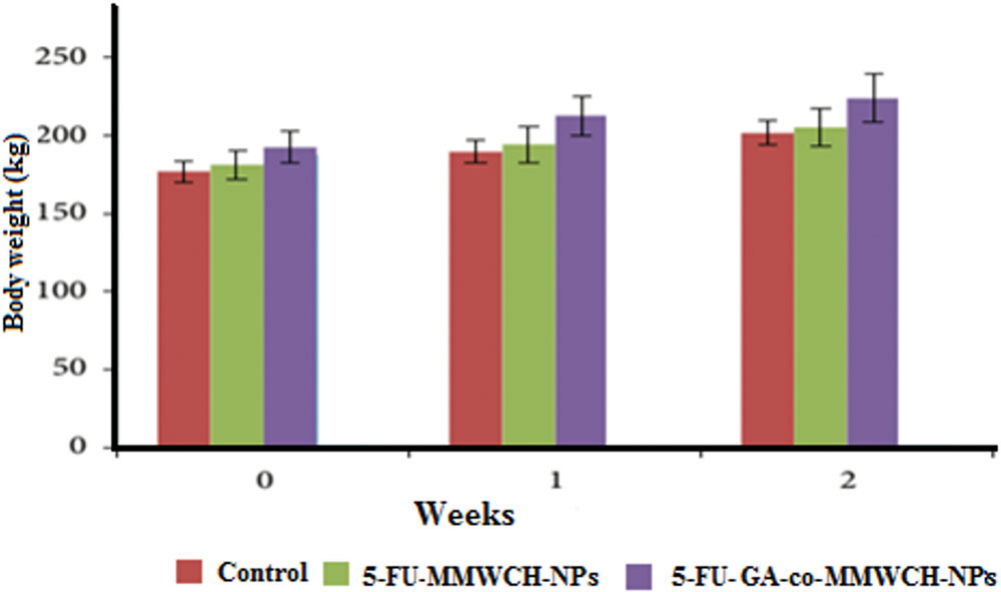
Mean body weight of rabbits receiving 5-FU loaded NPs after 14 days.

For evaluation of acute toxicity of our prepared and optimized nanoparticles formulations, histological slides of major organs of rabbits were prepared than were examined. Weight of rabbits and vital organs like stomach, heart, liver, spleen, kidney, and lung of rabbits (Koujitani et al., 1997; Olejniczak et al., 2001; Jaykaran et al., 2009) were measured before and after administration of control and prepared and optimized Nanoparticles formulations as body weight (kg), of vital organs of rabbits at 15th-day of study, are presented in Table 11 and Figure 8 respectively.

**Table 11.** Effect of oral dose (50 mg/kg) of optimized formulations on the organ weigh.

Treatment	Heart (g)	Liver (g)	Lung (g)	Kidney (g)	Stomach (g)	Spleen (g)
Control	3.60	60.1	9.72	12	12	1.1
Group II	4.0	64.0	9.50	11	11	13
Group III	4.2	66	10	10.8	12.7	1.1

### Hematology, lipid profile, and biochemical analysis

The vital organ of the body is blood so its different parameters were estimated in control as well as in both oral solution of developed carrier system 5-FU-MMWCH-NPs and 5-FU-GA cross-linked MMWCH-NPs treated rabbits & normal count was shown in all parameters of blood as acceptable limits of WBCs, RBCs, and platelets (Davis & Bredt, 1994; Slichter, 2004; Ljubimova et al., 2013) in Table 9. Lipid profiling parameters like urea, uric acid, cholesterol, triglycerides, and creatinine calculations (Malafaya et al., 2007; Lafontan & Langin, 2009; Shanks et al., 2009; Shayne, 2009) were normal and shown in Table 10. Biochemical analysis of all three groups of rabbits showed the same ranges of different parameters as all these values were within acceptable ranges. Our observations are similar to previous research work where they have conducted an acute toxicity study of acyclovir on mice and rabbits. Therefore, no-observable-adverse-effect-level (NOAEL) was calculated after oral administration of 10 g/kg of dose.

### Histopathological study

**Figure 9.**
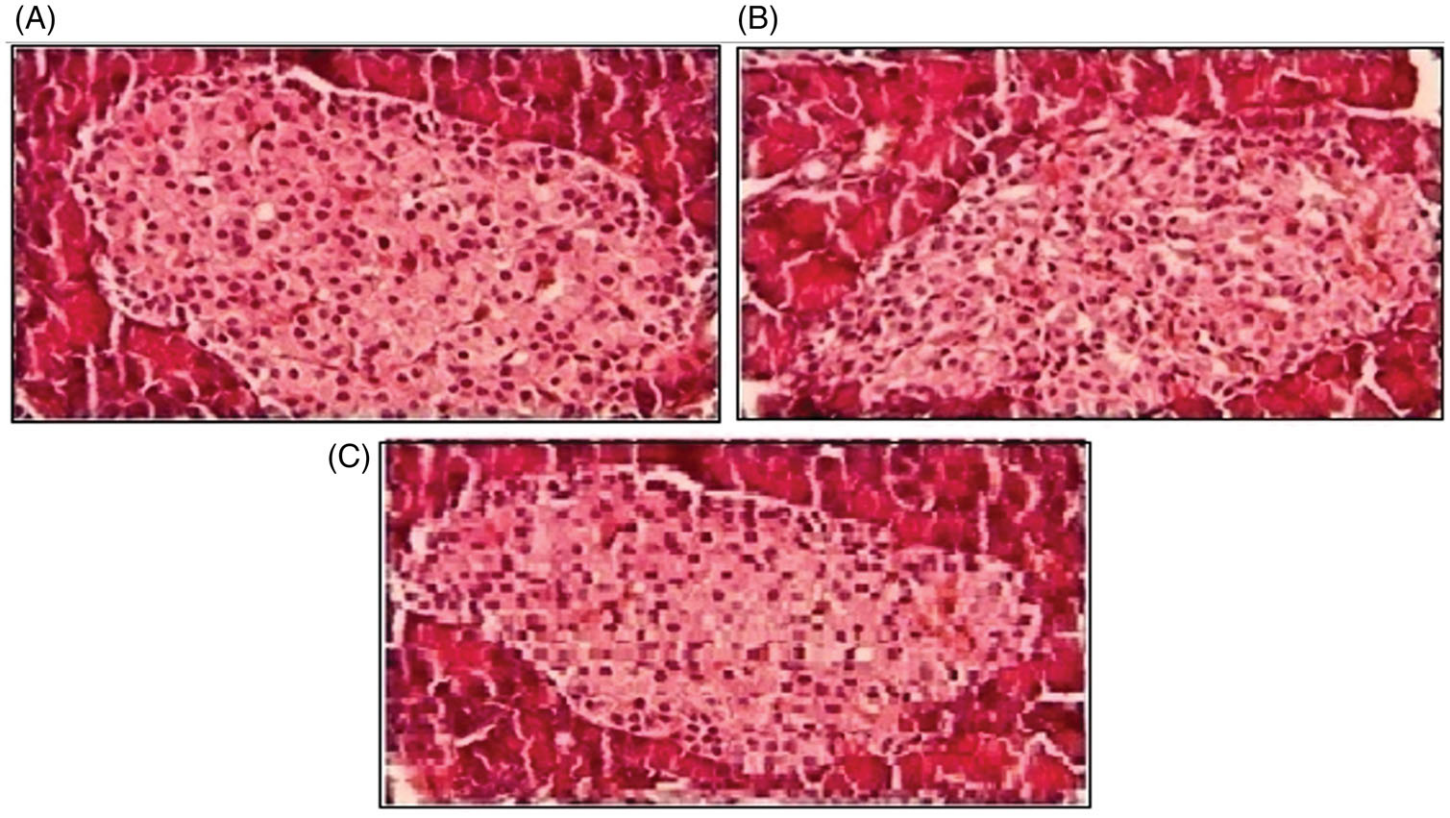
Histological slides of pancreas of rabbit of control group I (A), after oral solution of 5-FU-MMWCH-NPs treated group II (B) and 5-FU-MMWCH-NPs treated group III (C).

**Figure 10.**
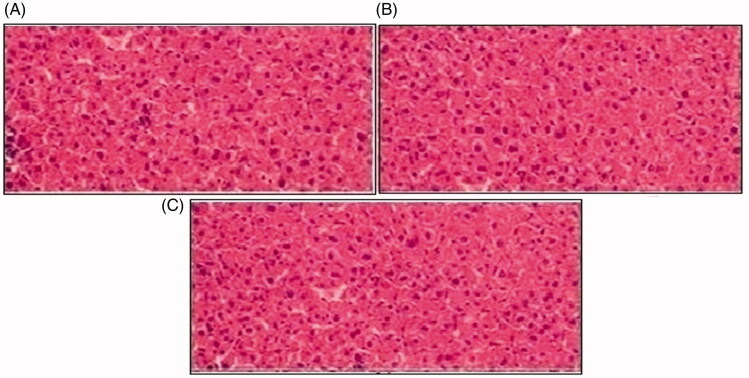
Histological examination of liver of rabbit of control group I (A), after oral solution of 5-FU-MMWCH-NPs treated group II (B) and 5-FU-MMWCH-NPs treated group III (C).

**Figure 11.**
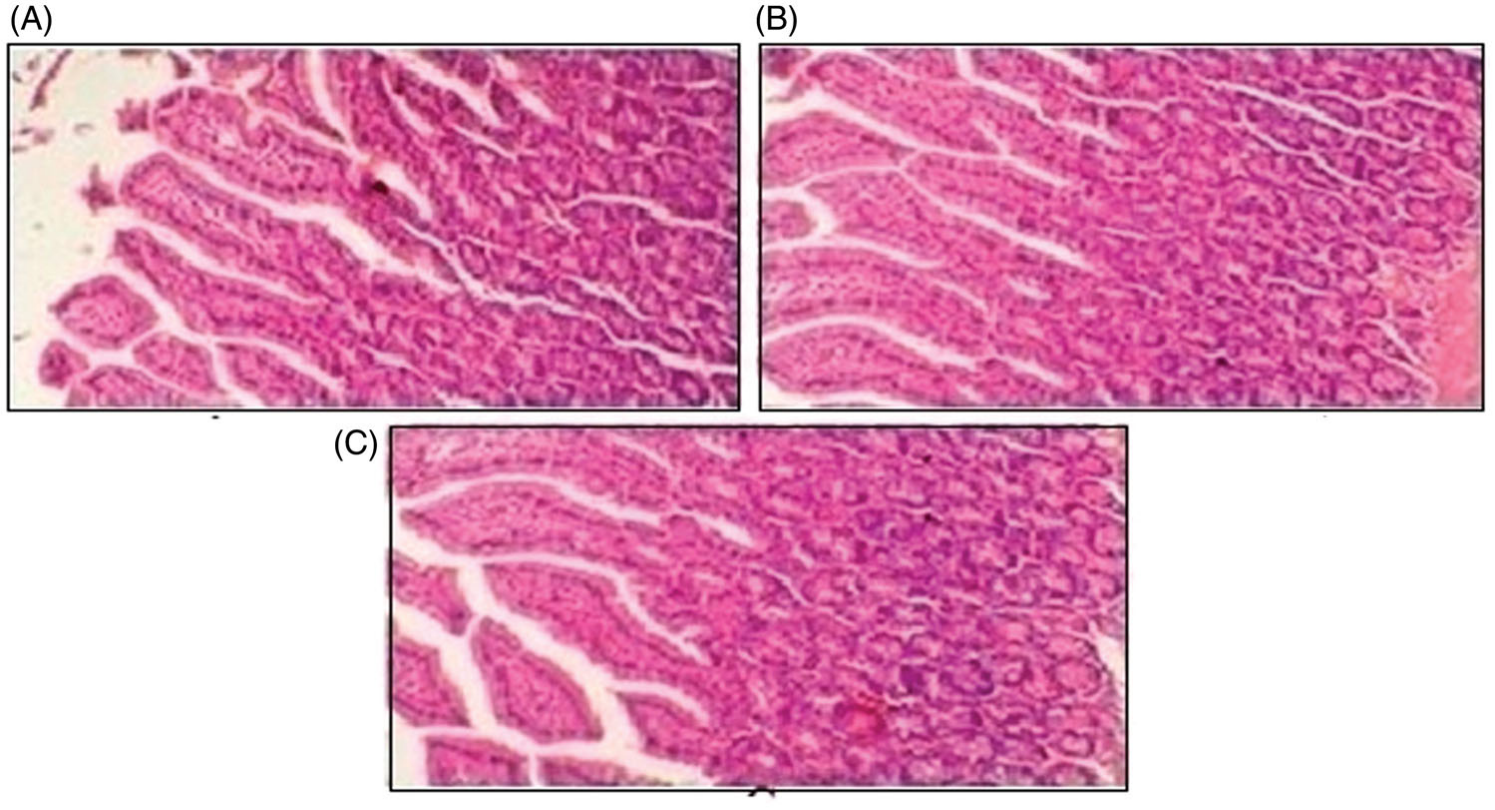
Histological slides of small intestine of rabbit of control group I (A), after oral solution of 5-FU-MMWCH-NPs treated group II (B) and 5-FU-MMWCH-NPs treated group III (C).

**Figure 12.**
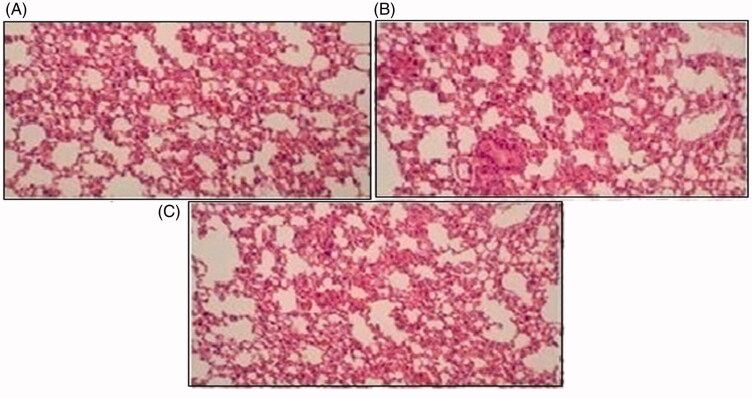
Histological slides of lung of rabbit of control group I (A), after oral solution of 5-FU-MMWCH-NPs treated group II (B) and 5-FU-MMWCH-NPs treated group III (C).

**Figure 13.**
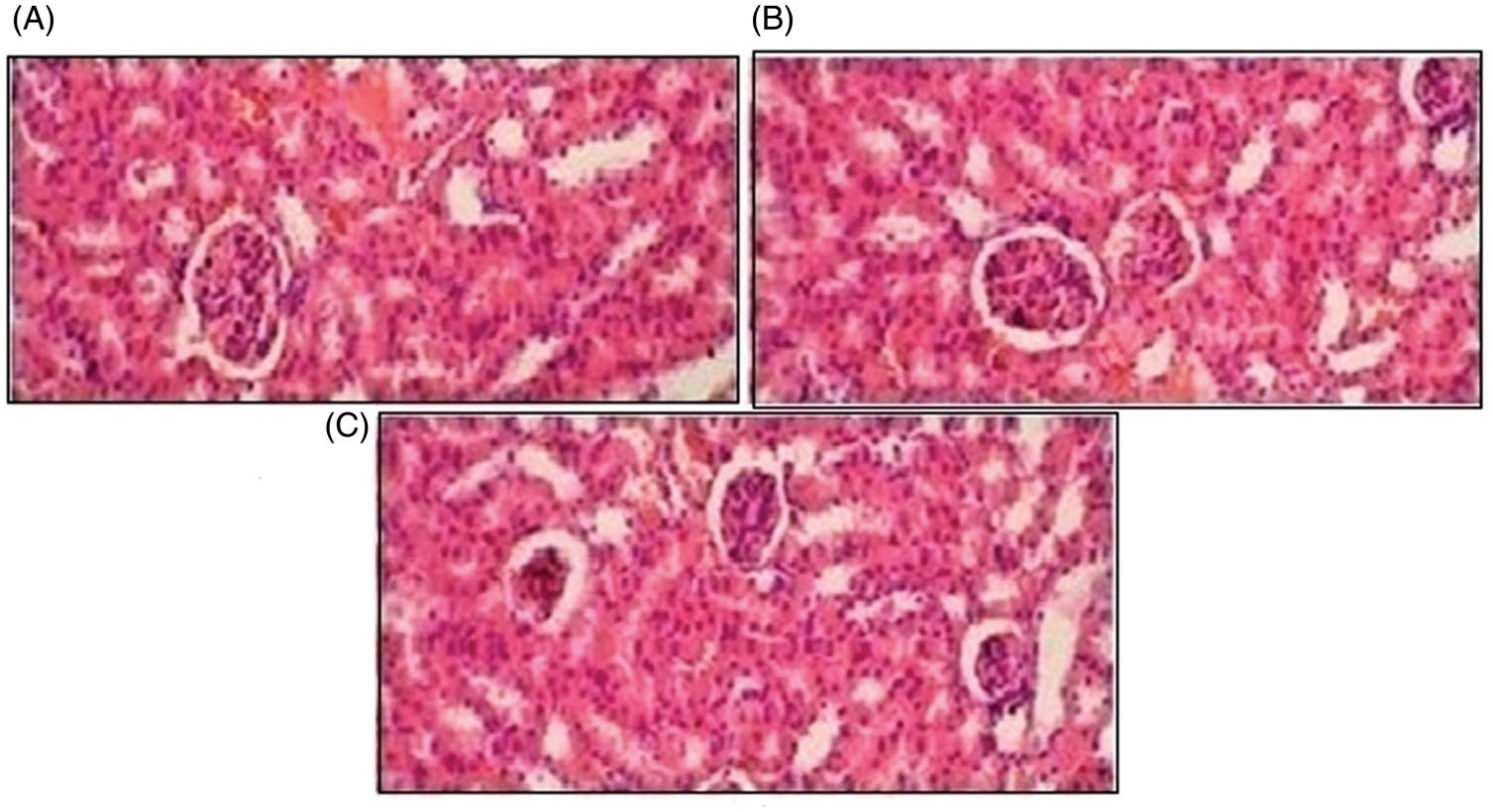
Histological slides of kidney of rabbit of control group I (A), after oral solution of 5-FU-MMWCH-NPs treated group II (B) and 5-FU-MMWCH-NPs treated group III (C).

**Figure 14.**
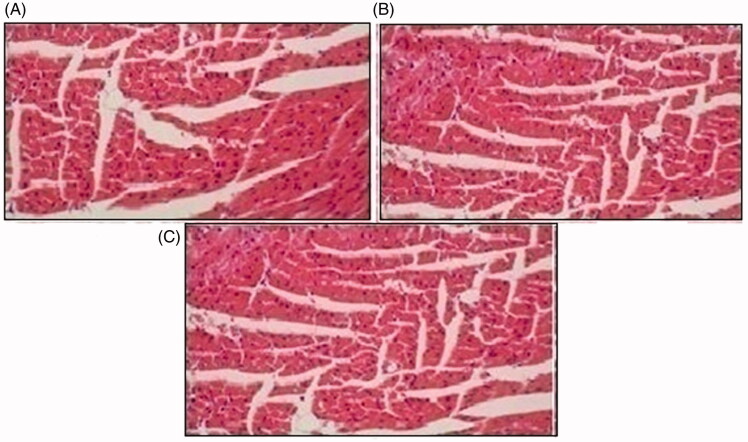
Histological slides of heart of rabbit of control group I (A), after oral solution of 5-FU-MMWCH-NPs treated group II (B) and 5-FU-MMWCH-NPs treated group III (C).

Histopathological slides of vital organs (heart, liver, kidney, lungs, and small intestine) of control, oral solutions of 5-FU loaded MMWCH-NPs and 5-FU loaded GA-co-MMWCH-NPs formulations under microscopic examination no clear cut lesions were shown in Figure 9 insignificant pathological variations were showed in islets of Langerhans of pancreatic tissues slides of rabbits (Shanks et al., 2009; Shayne, 2009). The normal lobular architecture was observed as in Figure 10 of the hepatic parenchyma slides of rabbits (Tan et al., 2016). No significant variations were observed in the small intestine slides sample of all rabbits (Thiagarajan et al., 2013) as shown in Figure 11. No significant pathology was present in lung slide samples of all rabbits as shown in Figure 12. Normal sizes of kidneys were observed in all sample slides of rabbits (United State Pharmacopeia-NF, 2016) as shown in Figure 13. Insignificant pathology of heart with normal myocardium slides samples of all rabbits (United State Pharmacopeia-NF, 2016; Organisation for Economic Co-operation, 2001a, 2001b; Piyasi et al., 2014) as shown in Figure 14. Thus, our prepared nanoparticle formulations with maximal tolerance dose were assessed to be higher than 50 mg/kg per day in rabbits.

## Conclusion

In this research work, an efficient drug encapsulation efficiency (%EE) and loading capacity (%LC) was attained which determined the strong interaction between polymer and drug depicts the effect of GA volume on the particle size of MMWCH-NPs, zeta potential (mV), drug entrapment efficiency (%EE) and drug loading capacity (%LC) respectively. Drug release from nano-carrier exhibited biphasic pattern with an initial fast release followed by the sustained release as compared with free 5-FU and 5-FU released from NPs was in accordance with Zero release order and then followed by Higuchi’s model with type II diffusion transport mechanism of drug release with Higuchi equation value (0.966). The therapeutic index of drugs (LD_50_/E D_50_), no mortality was observed in our studied rabbits even at highest doses till 14th-day of study. No toxicological changes were observed in body weight, feed consumption, ophthalmological examination, neurological-functional examination, hematology, clinical chemistry, and organ weight of albino rabbits.
